# Physicians' attitudes towards ePrescribing – evaluation of a Swedish full-scale implementation

**DOI:** 10.1186/1472-6947-9-37

**Published:** 2009-08-07

**Authors:** Lina Hellström, Karolina Waern, Emelie Montelius, Bengt Åstrand, Tony Rydberg, Göran Petersson

**Affiliations:** 1 eHealth Institute, School of Human Sciences, University of Kalmar, Kalmar, Sweden; 2 School of Pure and Applied Natural Sciences, University of Kalmar, Kalmar, Sweden; 3 Apoteket AB (National Corporation of Swedish Pharmacies), Stockholm, Sweden

## Abstract

**Background:**

The penetration rate of Electronic Health Record (EHR) systems in health care is increasing. However, many different EHR-systems are used with varying ePrescription designs and functionalities. The aim of the present study was to evaluate experienced ePrescribers' attitudes towards ePrescribing for suggesting improvements.

**Methods:**

Physicians (n = 431) from seven out of the 21 Swedish health care regions, using one of the six most widely implemented EHR-systems with integrated electronic prescribing modules, were recruited from primary care centers and hospital clinics of internal medicine, orthopaedics and surgery. The physicians received a web survey that comprised eight questions on background data and 19 items covering attitudes towards ePrescribing. Forty-two percent (n = 199) of the physicians answered the questionnaire; 90% (n = 180) of the respondents met the inclusion criteria and were included in the final analysis.

**Results:**

A majority of the respondents regarded their EHR-system easy to use in general (81%), and for the prescribing of drugs (88%). Most respondents believed they were able to provide the patients better service by ePrescribing (92%), and regarded ePrescriptions to be time saving (91%) and to be safer (83%), compared to handwritten prescriptions. Some of the most frequently reported weaknesses were: not clearly displayed price of drugs (43%), complicated drug choice (21%), and the perception that it was possible to handle more than one patient at a time when ePrescribing (13%). Moreover, 62% reported a lack of receipt from the pharmacy after successful transmission of an ePrescription. Although a majority (73%) of the physicians reported that they were always or often checking the ePrescription a last time before transmitting, 25% declared that they were seldom or never doing a last check. The respondents suggested a number of improvements, among others, to simplify the drug choice and the cancellation of ePrescriptions.

**Conclusion:**

The Swedish physicians in the group studied were generally satisfied with their specific EHR-system and with ePrescribing as such. However, identified weaknesses warrant improvements of the EHR-systems as well as of their implementation in the individual health care organisation.

## Background

For hundreds of years, the written prescription has been the method of choice for physicians to communicate decisions on drug therapy, and for pharmacists to dispense medications, while at the same time being a source for the patient about how to use the medication in order to maximize its benefit. Currently, the medical prescription is at the transitional stage between paper and the electronic state. When adapting a traditional process to the new electronic era, unique opportunities and challenges are offered the involved actors: patients, prescribers, pharmacists, and also health care and EHR-system providers and other stakeholders [1].

New technologies may introduce new risks with the extended use of the prescription information in large-scale databases [2,3]. A mistake by a prescriber may result in the incorrect medication being dispensed at the pharmacy, and also in the wrong documentation being recorded for future consultations. A mistake by a system programmer may also result in the incorrect medication being dispensed, but for a large group of patients. Future clinical decisions may also be based on wrong facts and assumptions, due to poor quality of the data provided. Thus, quality issues are even more accentuated in the electronic world [4].

The penetration rate of the Electronic Health Record (EHR) systems in Sweden is extensive, 86% of the employees in health care used EHR-systems in 2007 [5]. However, many different EHR-systems are employed, with varying ePrescription designs and functionalities. Even within a health care region or a single hospital, different EHR-systems are employed. Regardless of the EHR-system used, the prescriber must be confident with its functionality, effectiveness and safety.

**Figure 1:**
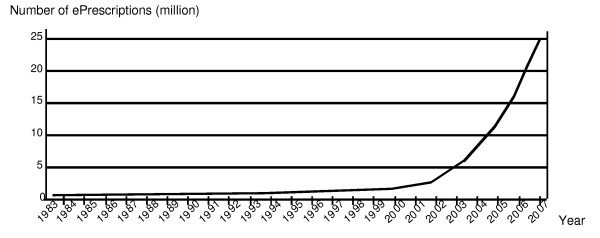
**Number of ePrescriptions in Sweden per year, between 1983 and 2007**. The first ePrescription was launched in 1983. After a new national strategy was decided at the end of the 1990's, the number of ePrescriptions in Sweden has escalated. Figure reproduced with permission [7].

The Swedish experience of ePrescribing began in 1983 with the world's first Electronically Transferred Prescription (ETP) for outpatients, between the computer systems in a doctor's office and a nearby outpatient pharmacy [6]. The first pilot project was followed by several others during the 1980's. After a new national strategy was decided at the end of the 1990's, the number of ePrescriptions in Sweden has escalated (Figure 1), with a penetration rate for ePrescriptions of about 70% of all new prescriptions in September 2008 [7]. More formal requirements (National ePrescription format, NEF) on the electronic communication process with automated quality checks are currently being implemented nationwide.

**Figure 2:**
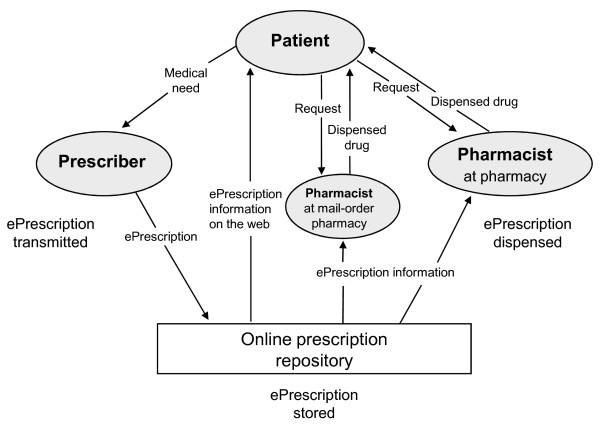
**Flowchart describing how ePrescriptions in Sweden are transmitted, stored and dispensed**. The patient contacts the prescriber because of a medical need. The prescriber transmits an ePrescription to the online prescription repository. The patient may then request the ePrescription by contacting a pharmacist at any pharmacy in Sweden or a pharmacist at a mail-order pharmacy. When dispensing, the pharmacist collects information about the requested ePrescription from the online prescription repository and may also store dispensing information. Patients can reach the online prescription repository directly, through the Internet, and get information about his/her ePrescriptions.

Figure 2 illustrates how ePrescriptions are transmitted, stored and dispensed in Sweden. An online prescription repository allows patients to store their prescriptions electronically for refills, and to have access to valid prescriptions (iterations included) at any pharmacy with the presentation of valid identification, making paper prescriptions obsolete. Patients may also have access to their stored prescriptions on the Internet by means of a secure digital authentication, enabling new services like mail-order delivery of prescription drugs.

The aim of the present study was to evaluate experienced ePrescribers' attitudes towards ePrescribing for suggesting improvements.

## Methods

### Setting and inclusion criteria

#### Selection of EHR-systems and health care regions

The six most widely used EHR-systems with integrated electronic prescribing modules in Sweden were included: Cosmic (version R7.1 sp 5 and R7.2 sp 2, Cambio Healthcare Systems), Journal III (version 1.83, Profdoc), Melior (version 212B, Siemens AB), SYSteam Cross (version 4.12.441 and 4.13.313, SYSteam), Take Care (version 10.1.1042, Profdoc) and VAS (version 3.2.02, TietoEnator). All the electronic prescribing modules were for the prescribing of drugs to outpatients and dispensing by community pharmacies. The six EHR-systems had a cumulative market share of 88.3% [5]. We identified seven out of the 21 health care regions in Sweden, which had implemented one of the six EHR-systems at least six months ago. The regions were geographically diverse and both sparsely-populated areas as well as metropolitan areas were represented. Of the total number of ePrescriptions in Sweden, 48% were prescribed in these seven health care regions. The penetration rate of ePrescriptions in the different regions varied between 50 and 86% (Table 1). Two of the EHR-systems were not used in primary care and one EHR-system was used only in primary care.

**Table 1:** ePrescription charateristics in the seven health care regions studied.

Health care regions included in study	ePrescriptions (%)	EHR-systems implemented
Norrbotten	86	F
Stockholm	83	B, E
Kronoberg	81	A
Blekinge	80	D
Uppsala	80	A
Västernorrland	54	D
Skåne	50	C
Median (IQR)	80 (15)	

The percentage of dispensed ePrescriptions of the total number of dispensed prescriptions in each studied health care region and Electronic Health Record (EHR) systems implemented in each health care region in October 2007 [Personal communication, E Ongwae, Apoteket AB].

A = Cosmic, B = Journal III, C = Melior, D = SYSteam Cross, E = Take Care and F = VAS

#### Selection of physicians

We recruited physicians from four disciplines; primary care, internal medicine, orthopaedics, and general surgery. The rationale for selecting these four disciplines was that we wanted to survey physicians prescribing a low number as well as a high number of ePrescriptions per day. Our assumption was that orthopaedics and general surgeons were rare users of the ePrescribing module, primary care physicians were frequent users and physicians from internal medicine were intermediate users. The total number of Swedish physicians working in Swedish health care in year 2006 was 32,495 [8]. About 40% were working in one of the four disciplines included in this study.

### Survey

A survey was developed with eight questions on background data and 19 items covering attitudes. The survey was piloted with four physicians. The response formats were a six-point response scale from "not agree at all" to "fully agree", multiple choice or free text. The forced six-point response scale was chosen over a five-point scale to avoid problems with less informative data and difficulties in interpretation of results, due to a considerable amount of answers in the midpoint response category [9]. A web based application for electronic surveys was employed [10]. The physicians received an e-mail with a web link to the survey together with information about the purpose of the study, who the investigators were, that the survey was voluntary and would take approximately 5–10 minutes to fill, and that the results would never be presented at an individual level, only at group level. No incentives were offered. The web survey was opened to be filled between September 5th and October 10th, 2007. Three reminders were sent during this time frame. The distribution of the web survey was based on lists of e-mail addresses provided by selected clinical heads. The clinic heads were instructed to provide a list of all, or at least 15 physicians, with no other specification of which physicians to choose.

**Figure 3:**
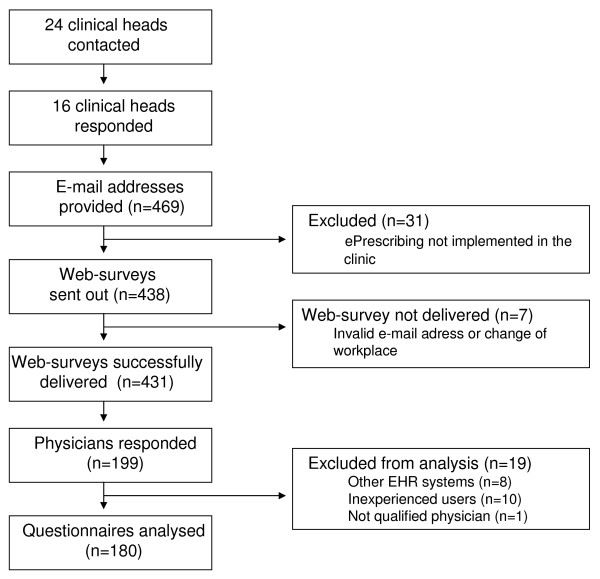
**Overview of the survey process of physicians**.

The survey process is illustrated in Figure 3. Forty-six percent (199/431) of the surveyed physicians responded to the questionnaire. The response rate for the final sample was 42% (180/431). There were 113 men (63%) and 67 women (37%) included in the study, which can be compared to all Swedish physicians where, in 2006, 57% were men and 43% women [8]. ePrescribing characteristics and the number of respondents from each discipline are presented in Table 2. Fifteen percent of the respondents had used an electronic system for two months to one year, and 85% for more than one year.

**Table 2:** Characteristics of respondents.

Respondents (n) per discipline and number of transmitted ePrescriptions per day, respectively, distributed per EHR-system (A-F)^a^.								
	A	B	C	D	E	F	Total n	(%)

*Discipline*^b^								
Primary care	6	22	-	9	-	26	63	(35)
Internal medicine	22	-	8	2	24	2	58	(32)
General surgery/	23	-	13	6	6	11	59	(33)
orthopaedics								
								
*Number of ePrescriptions per day*^c^								
< 5 "rare users"	26	-	12	7	12	6	63	(35)
5–10 "intermediate users"	15	11	9	3	10	9	57	(32)
> 10 "extensive users"	10	11	-	7	7	24	59	(33)
no response	-	-	-	-	1	-	1	(1)
								
*All*								
n	51	22	21	17	30	39	180	
(%)	(28)	(12)	(12)	(9)	(17)	(22)		

^a ^A = Cosmic, B = Journal III, C = Melior, D = SYSteam Cross, E = Take Care and F = VAS. ^b ^Completeness rate 1 (180/180). ^c ^Completeness rate 0.99 (179/180).

We consulted the regional research ethics committee, and filled in a form to inquire whether there was a need for revision and approval by the research ethics review board. The inquiry showed that a formal application to the research ethics review board was not necessary for this study.

### Statistics

The collected survey answers were analysed using Microsoft Office Excel 2003 (Microsoft Corp, Seattle, Wash.), Statistix 8 (Analytical Software, Fla.), and GraphPad Prism version 5.01 for Windows (GraphPad Software, San Diego California, http://www.graphpad.com). Survey answers were presented in absolute numbers and as part (%) of the respondents (n = 180). The part (%) of the respondents agreeing to the statements was calculated as number of respondents answering point 4–6 on the six-point scale divided by all respondents. Medians and corresponding interquartile ranges (IQR) were also reported. We examined the associations between the general attitudes (see statements in Table 3) and the time that the physician had been using his current ePrescription system (two groups), using Mann-Whitney U-test. The associations between the general attitudes and the extent of use of ePrescriptions (three groups) were analysed using Kruskal-Wallis test with Dunn's post-test. A p-value < 0.05 was considered significant. Free text answers were categorised and reported as absolute numbers and part (%) of respondents.

**Table 3:** Respondents' attitudes towards the ePrescribing module of their EHR-system.

Statement^a^	1	2	3	4	5	6	Don't know	total	median	IQR
The EHR-system being used at my workplace is easy to use.	4	12	18	35	68	43	0	180^b^	5	1
In the EHR-system that I use, it is easy to prescribe drugs.	4	7	11	24	46	88	0	180^b^	5	2
When ePrescribing drugs, the EHR-system clearly displays the personal security number of the current patient.	4	4	8	12	39	109	2	178^c^	6	1
When ePrescribing drugs, the EHR-system clearly displays the name of the current patient.	3	4	9	14	41	106	0	177^d^	6	1
When ePrescribing drugs, the EHR-system clearly displays the different packages for each drug.	7	11	18	28	41	72	0	177^d^	5	2
When ePrescribing drugs, the EHR-system clearly displays the price for each drug.	38	23	16	21	27	43	11	179^c^	4	4
Compared to handwritten prescriptions, ePrescriptions written in the EHR-systems are time saving.	2	4	6	10	29	125	2	178^c^	6	1
Compared to handwritten prescriptions, ePrescriptions written in the EHR-system are safer.	2	4	17	22	43	84	8	180^b^	5	2
Compared to handwritten prescriptions, I can offer my patients better service through ePrescriptions written in the EHR-system.	1	2	7	15	40	111	4	180^b^	6	1

Respondents' (n) degree of agreement to survey statements on a six-point scale.

^a ^Respondents were asked to agree/disagree to the statements on a six point scale where 1 = not agree at all and 6 = fully agree. Completeness rates ^b ^1.00, ^c ^0.99, and ^d ^0.98, respectively.

## Results

A majority of the respondents regarded their EHR-system easy to use in general (81%; 146/180) and for the prescribing of drugs (88%; 158/180) (Table 3). Most respondents believed they were able to provide the patients better service by ePrescribing (92%; 166/180), and regarded ePrescriptions to be time saving (91%; 164/180). Eighty-three percent (149/180) stated that ePrescriptions were safer than handwritten prescriptions. The different packages and the price were regarded clearly displayed by 78% (141/180) and 51% (91/180), respectively.

We found a difference in attitudes between physicians that were rare (< 5 ePrescriptions/day), intermediate (5–10 ePrescriptions/day) and extensive (> 10 ePrescriptions/day) users of ePrescribing. The intermediate and extensive users, to a greater extent than the rare users, regarded their EHR-system easy to use (p = 0.0003), easy to prescribe drugs with (p = 0.0001) and to be time saving (p ≤ 0.0001). The intermediate and extensive users, to a greater extent than the rare users, also regarded the patient's security number (p = 0.01) and name (p = 0.04), together with the price of the drug (p = 0.0002), to be clearly displayed. To the statements concerning safety and better service to the patients, extensive users agreed to a higher degree than intermediate and rare users (p = 0.01 and p = 0.0005, respectively). We found no differences in attitudes towards how the EHR-systems display different packages.

Respondents who had been using their EHR-system for ePrescribing more than one year, were more likely than respondents who had been using ePrescribing for less than a year, to regard their EHR-system easy to use (p = 0.004) and easy to prescribe drugs with (p = 0.004). They also, to a greater extent, regarded the patient's personal security number (p = 0.003) and name (p = 0.01) to be clearly displayed in their EHR-system when ePrescribing. However, there was no evidence that respondents who had been ePrescribing through their EHR-system for more than one year were more likely to regard ePrescriptions as time saving, safer or to perceive that they provided better service to patients. Also, we could not show any differences in attitudes concerning how the price and packages were displayed.

The respondents' opinions on strengths and weaknesses are presented in Table 4 and 5. The most frequently reported opinion of strengths with ePrescribing was 'fast and easy' (30%); the most frequently expressed weakness was 'complicated drug choice' (21%). Suggested improvements are reported in Table 6.

**Table 4:** Respondents' perceived strengths with ePrescribing

Question	Opinion^a^	n	%^b^
What strengths do you associate with the EHR-system that you use for ePrescribing? Describe the events or features you find particularly easy/good.^c^			
	Fast and easy	54	30
	Easy to renew prescriptions	38	21
	Gives a good overview, particularly the list of drugs prescribed to a patient within the health care region	23	13
	Information transfer between different features of the EHR-system, e.g. prescribed drugs are automatically archived in the patients health record	21	12
	The overview of patient's prescribed drugs	17	9
	Improved safety	16	9
	Easy access to the drug reference list (FASS)^d^	11	6
	Clear information on e.g. packaging and price	7	4
	Everything/the most	5	3
	Drug interaction alerts	4	2
	Other	20	11
	Total *(135 respondents)*	216	

^a ^Respondents expressed their opinions in free text answers which have been categorised. ^b ^Part (%) of the total number of respondents (N = 180). ^c ^Completeness rate 0.75 (135/180). ^d ^Pharmaceutical Specialties in Sweden.

**Table 5:** Respondents' perceived weaknesses with ePrescribing

Question	Opinion^a^	n	%^b^
What weaknesses do you associate with the EHR-system that you use for ePrescribing? Describe the events or features that you think are particularly hard/not so good.^c^			
	Drug choice is complicated	38	21
	No link between ePrescriptions and ApoDos/e-Dos^d^	21	12
	Complicated in general, e.g. many mouse clicks	20	11
	The cancel-/return function is inadequate	16	9
	Satisfied	10	6
	Several patients can be displayed at the same time	8	4
	ePrescription for extemporaneous preparations, non-approved drugs, and to patients seeking asylum is impossible/hard	7	4
	Do not get a receipt from the pharmacy	5	3
	Discontinuation of treatment is complicated	5	3
	The overview of the patient's prescribed drugs is disordered	5	3
	Insufficient warning of drug interactions	3	2
	No warning of too high doses	2	1
	Too easy	2	1
	Other	28	16
	Total *(139 respondents)*	170	

^a ^Respondents expressed their opinions in free text answers which have been categorised. ^b ^Part (%) of the total number of respondents (N = 180). ^c ^Completeness rate 0.77 (139/180). ^d ^Multi-Dose drug dispensing

**Table 6:** Respondents' suggestions for improvements of ePrescribing

Question	Opinion^a^	n	%^b^
These are my suggestions for improvements of ePrescriptions:^c^			
	Simplified drug choice	21	12
	Link with ApoDos/e-Dos^d^	21	12
	Cancellation of ePrescriptions possible	19	11
	Satisfied!	7	4
	eDialog between the prescriber and the pharmacy	7	4
	ePrescription for non-approved drugs and to patients seeking asylum	5	3
	Receipt from the pharmacy	4	2
	Graphical illustrations	3	2
	Possibility to save favourites	3	2
	Reduced mouse clicks	3	2
	Improved layout	2	1
	Improved information concerning drug interactions	2	1
	Other	32	18

	Total *(100 respondents)*	129	

^a ^Respondents expressed their opinions in free text answers which have been categorised. ^b ^Part (%) of the total number of respondents (N = 180). ^c ^Completeness rate 0.56 (100/180). ^d ^Multi-Dose drug dispensing

A minority of the respondents (13%; 23/180) perceived that it was possible to handle more than one patient at a time when ePrescribing. A majority (73%; 132/180) reported that they were always or often performing a final check of the ePrescription before transmitting; 25% (45/180) declared that they were seldom or never performing a final check.

**Figure 4:**
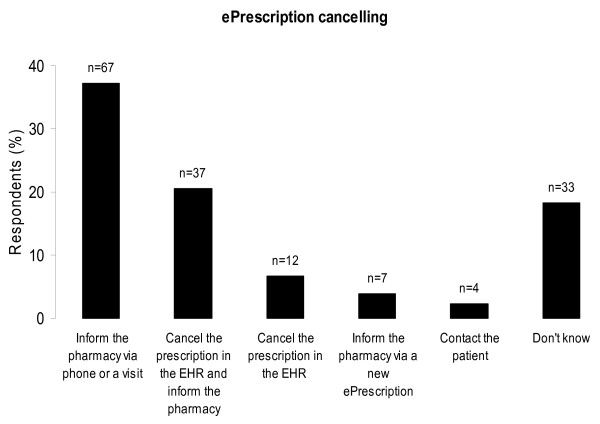
**Respondents' procedures when cancelling prescriptions**. Respondents' answers to the question *"Describe your actions when/if you want to cancel an ePrescription after the prescription is transmitted to the pharmacy." *The respondents (%) on the y-axis and number of respondents (n) on top of the columns. The completeness rate was 0.89 (160/180).

**Figure 5:**
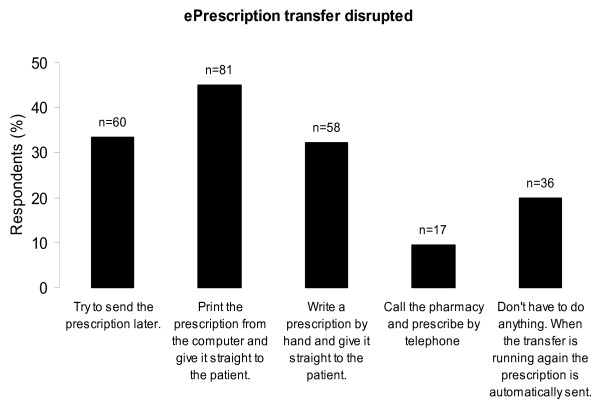
**Respondents' procedures when the ePrescription transfer is disrupted**. Respondents' answers to the question *"How do you proceed if/when you get a message that the ePrescription transmitting process is down, that is, it is not possible to transmit ePrescriptions? The patient is with you and is not in acute need of the drug"*. The respondents (%) on the y-axis and number of answers (n) on top of the columns. One respondent could give more than one answer. The completeness rate was 1.00 (180/180).

The routines of cancelling prescriptions and of handling the situation when the ePrescription transfer is disrupted showed great variability (Figure 4 and 5). This variability could also be observed within the different EHR-systems. The answers to the question 'After sending an ePrescription to the pharmacy, do you get a receipt from the pharmacy that the ePrescription is received?' differed; 62% (111/180) reported they did not get a receipt, 26% (47/180) reported they got a receipt, and 12% (21/180) did not know.

## Discussion

The group of Swedish physicians studied was generally satisfied with their specific EHR-system and with ePrescribing as such. However, we identified some problems that may affect the ePrescribing process, e.g. one quarter of the physicians seldom or never performed a final check of the ePrescription before transmission, the drug choice was perceived to be complicated, and there were different routines among physicians when cancelling ePrescriptions or in case of disrupted ePrescription transmission. Also, more than half of the respondents reported a lack of receipt from the pharmacy after successful transmission of the ePrescription. These weaknesses in the ePrescribing process warrant improvements of the provided EHR-systems per se, as well as of their implementation in the individual health care organisation, and the associated training for users.

The present study was not designed to quantify differences or to rank the six different EHR-systems. Rather, it was useful to identify general attitudes among users, and also to reveal strengths and weaknesses in the ePrescribing processes. We regard our study to reflect the situation for the Swedish physicians in 2007 in the selected health care regions but not to be representative for all physicians, since the population included was selected and thus not a random sample. However, physicians from 7 out of 21 health care regions and 4 different disciplines were included. Furthermore, the six EHR-systems included in the study dominate the Swedish market. We cannot exclude that attitudes and behaviour differ from other health care regions depending on the EHR-system used, the organisation, and how the prescribers are trained and supported.

The results should be regarded as hypothesis generating, depending on the non-random method of recruitment. Our selections of regions and clinical heads as well as the clinical heads' ways to provide us with e-mail addresses represent potential risks for selection biases. A random sample of physicians and a higher response rate would have improved the validity of the study. In contrast to many other countries, Sweden has a single-payer health care system which might result in a less complex ePrescribing process. Consequently, the generalisability of the results to other nations might be limited.

We did not collect data about how long respondents had used handwritten prescriptions, and we cannot exclude that some respondents had never used handwritten prescriptions. However, given that the ePrescribing systems have been implemented at one clinic at a time, mainly during the last five years, and physicians work at a number of different clinics during their education and further training, we estimate the probability that a substantial proportion of the respondents had never used handwritten prescriptions as small.

A majority of the respondents stated that ePrescriptions are time saving, in agreement with previous studies [1,11,12]. A study using time-motion techniques to compare prescribing times for paper-based prescribing and ePrescribing reported no differences in prescribing times [13]. In our study, more than 90% of the physicians felt they were able to offer their patients better service by ePrescribing. An example of the improved service according to the respondents is that patients are able to fill the prescription at any Swedish pharmacy.

The varying answers to the questions about cancelling prescriptions and disrupted ePrescription transmission might be explained by the lack of distinct routines and training. When cancelling prescriptions, prescribers should always inform the pharmacy and cancel the prescription in the EHR. How to proceed when the ePrescribing transmission is disrupted depends on which EHR-system the prescriber is using. In some EHR-systems, the prescription is automatically sent when the transfer is restored, in others this function is missing. The prescriber needs to be aware of how disrupted ePrescribing transmission is managed in his/her specific EHR-system; otherwise the result could be duplicate prescriptions or no prescription at all. The finding that 13% of the physicians perceived it possible to handle more than one patient at a time was hard to interpret, as all EHR-vendors state that this is impossible.

For some of the survey items, rare users of ePrescriptions were found to have more negative attitudes and extensive users more positive attitudes towards ePrescribing. Physicians transmitting more than five ePrescriptions per day were more positive towards ePrescribing than those prescribing less with respect to ease of use, time savings, and how clearly the patient's personal security number and name together with the price of the drug were displayed. A similar pattern was seen when analysing differences between prescribers who had used their EHR-system for less than a year and prescribers who had used it for more than a year; the more inexperienced users being more negative about ease of use and how the personal security number and name were displayed. One explanation to the differences in attitudes between these groups might be that physicians have to use the ePrescribing function regularly and extensively in order to fully appreciate it [14,15]. The ePrescribing function of the EHR-systems might not be intuitive enough for being fully appreciated also by rare users.

ePrescriptions were considered as safer compared to handwritten prescriptions by the majority of the respondents; an opinion supported by others [11,16-19]. However, prescribers and pharmacists need to be aware that EHR-systems may introduce new types of errors [16,18,20]. We revealed important weaknesses, which could be attributed to the user interface, special features in the EHR-systems or lack of distinct routines. None of the EHR-systems investigated was "perfect" overall. Some of these shortcomings might imply an increased risk for medication errors. Based on our findings, we suggest that:

- The prescriber should get a receipt when the transmission is complete, and know how it is displayed.

- It should be mandatory to check the ePrescription a last time before transmitting it to the pharmacy.

- There have to be distinct routines how to handle situations when the ePrescription transfer is disrupted.

- The EHR-system must clearly and unambiguously display the patient's name and personal security number.

To improve the ePrescribing process, there is a need for extensive guidelines and distinct requirements from the authorities. There is also a need for good cooperation between EHR-system software providers and health care providers to enable compliance to these guidelines. We believe that it is necessary to have a more formal certification procedure of EHR-systems and ePrescribing.

Since ePrescription during the last few years has developed to be the regular way to prescribe drugs in Sweden, repeated user studies are warranted aiming at further improvement of quality and security of the ePrescription process. We also recommend research studies for investigation of new types of prescribing errors, including risk assessment for patients.

## Conclusion

The Swedish physicians in the group studied were generally satisfied with their specific EHR-system and with ePrescribing as such. However, identified weaknesses warrant improvements of the EHR-systems as well as of their implementation in the individual health care organisation.

## Competing interests

The authors declare that they have no competing interests.

## Authors' contributions

KW, GP and BÅ designed the study and developed the survey. KW was responsible for collecting survey answers and for the initial analysis of the survey answers. EM and LH were responsible for the figures and tables, and conducted the statistical analysis. All authors participated in the interpretation of data. LH, BÅ and TR drafted the manuscript. GP, LH, BÅ, EM and TR made critical revisions to the manuscript. All authors gave final approval to the manuscript.

## Pre-publication history

The pre-publication history for this paper can be accessed here:

http://www.biomedcentral.com/1472-6947/9/37/prepub
